# Bioavailable iron in the Southern Ocean: the significance of the iceberg conveyor belt

**DOI:** 10.1186/1467-4866-9-7

**Published:** 2008-05-30

**Authors:** Rob Raiswell, Liane G Benning, Martyn Tranter, Slawek Tulaczyk

**Affiliations:** 1 Earth and Biosphere Institute, School of Earth and Environment, University of Leeds, Leeds, LS2 9JT, UK; 2 Bristol Glaciology Centre, School of Geographical Sciences, University of Bristol, Bristol, BS8 1SS, UK; 3 Department of Earth and Planetary Sciences, University of California, Santa Cruz, CA 95064, USA

## Abstract

Productivity in the Southern Oceans is iron-limited, and the supply of iron dissolved from aeolian dust is believed to be the main source from outside the marine reservoir. Glacial sediment sources of iron have rarely been considered, as the iron has been assumed to be inert and non-bioavailable. This study demonstrates the presence of potentially bioavailable Fe as ferrihydrite and goethite in nanoparticulate clusters, in sediments collected from icebergs in the Southern Ocean and glaciers on the Antarctic landmass. Nanoparticles in ice can be transported by icebergs away from coastal regions in the Southern Ocean, enabling melting to release bioavailable Fe to the open ocean. The abundance of nanoparticulate iron has been measured by an ascorbate extraction. This data indicates that the fluxes of bioavailable iron supplied to the Southern Ocean from aeolian dust (0.01–0.13 Tg yr^-1^) and icebergs (0.06–0.12 Tg yr^-1^) are comparable. Increases in iceberg production thus have the capacity to increase productivity and this newly identified negative feedback may help to mitigate fossil fuel emissions.

## Background

Iron is believed to limit phytoplankton productivity in the Southern Ocean [1-3], where insufficient Fe is supplied by upwelling from deep waters to surface waters to allow phytoplankton to utilise all the nitrate. Hence any additional Fe sources to the surface waters of the Southern Ocean have the potential to stimulate extra primary productivity [1,3,4]. Most research to date has focused on the addition of Fe to surface waters by dissolution from aeolian dust [2-5]. Other important but more localised sources are derived from melting sea ice [6,7] and the resuspension of shelf sediments [6,8]. However recent work indicates that Fe derived from iceberg-hosted sediment may be an important, and hitherto unrecognised, source of bioavailable Fe [9,10]. These findings are significant because the input of icebergs to the Southern Ocean is increasing [11] and any accompanying productivity increase has the potential to enhance carbon export and provide a new negative feedback loop to mitigate fossil fuel emissions (but see [3,12]).

Potential bioavailable inputs from glacial sediment have been overlooked, primarily because Fe in these sediments was assumed to be too inert for plankton to utilise. Two recent studies present a contrasting view. Firstly, high resolution microscopy [9] has shown that sediments (mainly proglacial and supraglacial) associated with glaciers (in Antarctica and elsewhere) contain nanoparticulate iron oxyhydroxides (~5–10 nm diameter). Only a small fraction of the iron oxyhydroxide pool is present as nanoparticulates, but this fraction is biogeochemically reactive and potentially bioavailable. This study [9] only reported on one sample of glacial sediment enclosed in ice and did not attempt to quantify the nanoparticulate Fe contents (although measurements of Fe present in the whole oxyhydroxide pool indicated that <<1% of this pool need be nanoparticulate and bioavailable to fertilise a significant increase in productivity). Further studies of sediments enclosed in ice are needed, because icebergs can transport such sediments away from coastal regions to fertilise productivity in the open ocean. Secondly, melting icebergs in the Weddell Sea [10] are associated with hot spots of biological activity and it is suggested that enhanced productivity was caused by the release of terrigenous debris that apparently supplied bioavailable Fe. Significant enrichments of glacial sediment around two icebergs (> 0.1 km^2 ^in area) were found, as were high concentrations of chlorophyll, krill and seabirds. Extrapolation of these results to the Weddell Sea as a whole [10] suggests that similar-sized icebergs already influence biological activity in over 39% of the surface ocean in this area. Furthermore iceberg-hosted sediment was demonstrated to stimulate productivity in experiments carried out under Fe-limited conditions, such as those existing in the Southern Ocean [10]. These studies [9,10] suggest that iceberg-hosted sediment has the potential to stimulate significant primary productivity in the Southern Ocean.

In this contribution we present data on sediment and icemelt composition from icebergs collected at two different locations in the Southern Ocean and from two glaciers in Antarctica. High resolution microscopy studies of sediments collected by melting these samples demonstrate the presence of nanoparticulate Fe oxyhydroxides. We utilise a buffered ascorbate solution to extract nanoparticulate Fe oxyhydroxides and thus estimate the concentrations of potentially bioavailable Fe in iceberg-hosted and glacial sediment. Finally we recalculate global estimates [9] of the Fe oxyhydroxide fluxes derived from icebergs, and other clastic and dissolved sources, to show that icebergs are a major source of bioavailable Fe to the Southern Ocean.

## Sampling and methodology

Four samples (S1-S4) were collected from icebergs driven on to the shores of Seymour Island (lat. 64° 18.9' S, long. 56° 46.7' W) from the Weddell Sea. Two icebergs (KG1 and KG2) were sampled from Admiralty Strait (lat. 62° 13.2' S, long. 58° 47.4' W), off King George Island. All the iceberg samples were collected from sediment-bearing layers in dense, clear blue ice representing compressed glacier ice rather than accreted frozen seawater (see later). Ice was chipped with a forged, hardened steel geological hammer, cleaned with alcohol. The outer layers of ice that might be contaminated were allowed to melt before the remaining ice was transferred into a new polyethylene bag and allowed to melt. Pebbles (> 1 mm diameter) were removed to prepare as thin sections. The sediment was allowed to settle before the meltwater was decanted off and filtered through 0.2 μm polycarbonate membrane filters pre-washed with sample. A similar procedure was followed for ice sampled from Taylor Glacier (lat. 77° 44' S, long. 162°10' E) and Canada Glacier (lat. 77° 36' S, long. 162°59' E). In both cases, slabs of basal sediment-bearing ice were collected in the field and sub-sampled in the laboratory with a tempered and hardened carbon steel saw cleaned in ethanol. Taylor Glacier provided three samples; T1 from a sediment-poor layer and T2 and T3 from different layers of basal, sediment-rich ice. The sample from Canada Glacier (C1) was also from basal, sediment-rich ice. The outer layers of ice were allowed to melt before the residual ice was melted through a Whatman 542 filter paper (pore size 2.7 μm; pre-washed with sample) to collect the sediment. This filtrate was then suction-filtered through a 0.2 μm polycarbonate membrane filter also pre-washed with sample. A pre-filtration step minimized melt-sediment contact times (and thus the potential for nanoparticle formation) in these sediment-rich samples. The filtered meltwaters were acidified (2% HNO_3_) and stored at 4°C in acid-cleaned glass vials until analysis by a Perkin Elmer Elan DRCe Inductively Coupled Plasma Mass Spectrometer. Detection limits were 0.02 nM.

The coarse-filtered iceberg samples contain a range of grain-sizes (up to silt- and sand-size), and this heterogeneity makes it difficult to sample representatively. Duplicate samples were therefore taken from the larger iceberg samples (S1, S4, KG1 and KG2) to provide a rough estimate of sampling variations. Each sample of air-dried, iceberg and glacial sediment was treated for 24 hours by an ascorbate solution buffered at pH 7.5, which extracts amorphous and nanoparticulate Fe oxyhydroxides [13]. The extractant solution was a deoxygenated solution of 50 g L^-1 ^sodium citrate and 50 g L^-1 ^sodium bicarbonate to which 10 g L^-1 ^of ascorbic acid was added. About 30 mg of sample were mixed with 10 ml of the ascorbate solution, shaken for 24 hrs at room temperature and then filtered through a 0.2 μm polycarbonate membrane filter. The Fe removed by ascorbate is hereafter termed FeA. The residual sediment was treated for 2 hrs with a solution of 50 g L^-1 ^sodium dithionite in 0.35 M acetic acid and 0.2 M sodium citrate, buffered at pH 4.8 [14]. Dithionite-soluble Fe is hereafter termed FeD. Both the FeA and FeD extractant solutions were analysed for Fe using a Varian Spectra AA-10 Atomic Absorption Spectrometer with an air-acetylene flame. Replicate analysis of a stream sediment standard gave precisions of 3% for FeA and 10% for FeD using this sequential extraction. Blank corrections were negligible.

For microscopic imaging and qualitative elemental analyses, dried samples were placed on an aluminium stub, then coated with a 3 nm platinum layer and analyzed using a Field Emission Gun Scanning Electron Microscope (FEG-SEM, LEO 1530) equipped with an Oxford Instruments energy dispersive X-ray (EDX) detector and INCA software. Images were collected at 3 kV and a working distance of 4 mm, while for EDX analysis a working distance of 8 mm and an accelerating voltage to 15 kV were used. Quantitative nanodiffraction and elemental analyses of selected samples were carried out using a Philips CM200 Field-Emission Gun -Transmission Electron Microscope (FEG-TEM) equipped with an Oxford Instrument UTW Energy Dispersive X-ray spectrometer (EDS) and selected area diffraction (SAED) capabilities. The dried samples were re-suspended in ethanol by ultrasonication and deposited on standard holey carbon support films on copper grids (Agar Scientific Ltd) and imaged at 197 keV.

## Results

Pebbles from the icebergs S1, S2, KG1 and KG2 contained a variety of lithologies including slates, and altered intermediate to acid igneous rocks (tonalite, granodiorite, dacite and rhyolite) that are typical of rocks from the Antarctic Peninsula (Leat, pers. comm.). The bedrock below the Canada and Taylor Glaciers is believed to be granite, dolerite and/or sandstone [9]. Concentrations of dissolved Fe in the iceberg melts are variable (0.05–3.8 nM) and show no correlation with sediment Fe contents (Table 1). Previous studies of the dissolved Fe released from glacier and sea-ice report concentrations typically in the range of 5 to 50 nM [6,15-17], which were suggested to originate either from aeolian dust or from sea-ice formed in coastal waters with relatively high dissolved Fe [6,7]. Our icemelt data are at the lower end of this range and are clearly derived from glacier ice because concentrations of chloride are low (< 0.1 ppm except for 20 ppm in T1).

**Table 1:** Composition of icemelt and sediment from Antarctic icebergs and glaciers.

**Sample**	**Icemelt**	**% Fe extracted from Sediment**	
**Icebergs**	**Dissolved Fe (nM)**	**Ascorbate (%FeA)**	**Dithionite (%FeD)**

S1 (Duplicates)		0.104 0.038	0.88 0.67
Mean	0.65	0.071	0.78
S2	0.16	0.195	0.86
S3	N/A	0.357	1.20
S4 (Duplicates)		0.19 0.104	0.95 0.66
Mean	0.30	0.15	0.81
KG1 (Duplicates)		0.071 0.042	0.33 0.27
Mean	2.2	0.057	0.30
KG2 (Duplicates)		0.057 0.059	0.42 0.84
Mean	0.13	0.058	0.63
**Iceberg Mean**	**0.69 ± 0.87**	**0.15 ± 0.12**	**0.76 ± 0.29**
**Glaciers**			
T1	0.05	0.029	0.14
T2	0.09	0.020	0.10
T3	3.8	0.029	0.10
C1	2.0	0.023	0.27
**Glacier Mean**	**1.5 ± 1.8**	**0.023 ± 0.004**	**0.15 ± 0.05**
**Iceberg+Glacier Mean**	**1.0 ± 1.3**	**0.10 ± 0.11**	**0.52 ± 0.39**

N/A Not available.

The data in Table 1 show that concentrations of Fe extractable by ascorbate (FeA) in the duplicate iceberg sediments mostly vary by a factor of 3 or less (e.g. S1 0.104% and 0.038%; S4 0.19% and 0.104%; KG1 0.071% and 0.042%; KG2 0.057% and 0.059%) but these differences are within the range of the between iceberg variation (0.06 to 0.36%). The iceberg sediments in Table 1 contain a mean of 0.15 ± 0.12% FeA whereas rather lower concentrations of FeA (0.023 ± 0.004%) occur in the glacial sediments, probably reflecting different source area lithologies (see above). Overall the mean of the iceberg and glacier samples is 0.10 ± 0.11% FeA. This extraction [13] mainly dissolves amorphous Fe oxyhydroxides and variable proportions of ferrihydrite depending on crystallinity, ageing and drying (> 97% of fresh 6 line nano-ferrihydrite, 76–97% of aged 6 line nano-ferrihydrite, 25–50% aged and freeze-dried 6 line nano-ferrihydrite and 3–25% aged and freeze dried 2 line nano-ferrihydrite). Since all ferrihydrite is nanoparticulate [18,19], we will subsequently only use the 'nano' prefix to distinguish nanoparticulate goethite from macrocrystalline goethite.

We have also found that ascorbate extracts small or negligible concentrations of Fe from other synthetic [20] Fe oxyhydroxide minerals (lepidocrocite 6.0%, goethite 0.6%, magnetite 0.0% and hematite 0.09%), consistent with pH 8 ascorbate extractions [21]. There are no other measurements of FeA on glacial sediments but measurements on intertidal and saltmarsh sediments are generally about an order of magnitude larger [13,21].

Table 1 shows that the subsequent extraction by dithionite on average dissolves 0.76 ± 0.29% FeD from the iceberg samples and 0.15 ± 0.05% FeD from the glacial sediments; a difference which is again probably related to the different lithologies in the source areas. Consistent with this, FeA and FeD are well-correlated (r = 0.87). The overall mean of the iceberg and glacial samples is 0.52 ± 0.39% FeD. This extraction dissolves 100% of the total Fe in lepidocrocite, ferrihydrite, goethite and hematite, and about 5–10% of the total Fe in sheet silicates [14,20]. Measurements of dithionite-extractable Fe (not preceded by an ascorbate extraction) for glacial sediment from Antarctica and elsewhere [22,23] have a mean of 0.47 ± 0.37% which is not significantly different from the sum of FeA and FeD in Table 1. Thus we believe that the sediment composition data in Table 1 are typical of glacial sediments.

**Figure 1:**
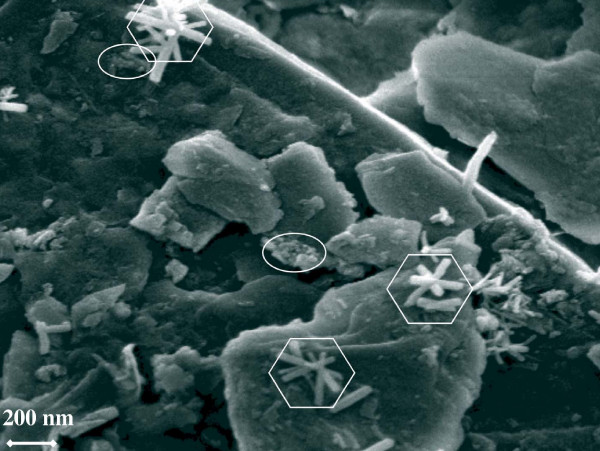
**Nanoparticulate Fe oxyhydroxides in iceberg sample S2, Seymour Island, Antarctica. **Nanoparticulate goethite rods are prominent in the octagonal areas, while the ellipses contain rubbly, ferrihydrite nanoparticulates. Both goethite and ferrihydrite were confirmed by high-resolution TEM imaging and nano-diffraction analysis (SAED). The platey morphologies represent the aluminosilicate substrates with which the Fe-nanoparticulates are usually associated.

High resolution microscopy shows that the iceberg and glacial sediments all contain nanoparticulate iron oxyhydroxides which commonly occur in two different morphologies; as rubbly aggregates (Fig. 1) up to several hundred nm in diameter, and as laths typically 100–200 nm in length and 20–40 nm in diameter that often occur as star-shaped aggregates (Fig. 1). EDS analysis confirms that both aggregates are Fe-rich and SAED demonstrates that the rubbly aggregates are ferrihydrite or nano-goethite, but the laths are always nano-goethite.

## Discussion

### Iron Oxyhydroxide Nanoparticulates: Origin and Bioavailability

The photograph in Fig 1 (see also [9]) clearly shows that iceberg and glacial sediments from Antarctica contain aggregates of ferrihydrite and nanogoethite. All the sediment samples in Table 1 have similar nanoparticulates, along with lesser amounts of nanoparticulates with different morphologies (as yet unidentified). In general nanoparticulates result from biogeochemical processes that generate high degrees of supersaturation and consequently many crystal nuclei [24]. It is likely that chemical weathering dissolves Fe from minerals in glacial debris either before transport, in subglacial environments where water is present, or during iceberg transport, where ice melting ice can interact with iceberg-hosted sediment (see later). High degrees of supersaturation may result from freezing and melting cycles. The nanoparticulate aggregates in S1-4, KG1-2, T1-3 and C1 all appear to be associated with aluminosilicate minerals. Aggregation and attachment may have been induced during sample preparation [9] but is also likely following release into the sediment-rich water surrounding melting icebergs [10]. Attachment to coarse sediment grains will significantly increase settling rates, and decrease the time for nanoparticulate Fe to be released into surface waters.

Attachment is likely following release into icemelt due to electrostatic attraction between nanoparticulates and aluminosilicates. Aluminosilicates generally possess an overall negative surface charge, due to isomorphous substitution of Si and Al by less positive valence ions and to the pH-dependent charges arising from surface hydroxyls, but there are also sites of positive charge, where lattice discontinuities occur at grain edges and surface irregularities [25]. Aluminosilicates thus possess sites with positive and negative surface charge characteristics and Fe nanoparticulates probably display a similar variability. The point of zero charge (the pH below which the surface charge is positive) of pure iron oxyhydroxides is variable (ferrihydrite 7.8–7.9 and goethite 7.5 to 9.5) and surface charge may be considerably modified by adsorbed ions, organic matter and by non-stoichiometric compositions [26]. Thus positive or negative surface charges are both possible in glacial meltwaters that in general range in pH from 6.5 to 8.5 [27]. The abundance of aluminosilicate grains in the iceberg sediments makes their close approach, collision and attraction to nanoparticles very probable. Once attached to sediment grains nanoparticulates may still be bioavailable either directly or indirectly (see below). However silt-sized grains (up to 50 μm diameter) will sink through 200 m of surface seawater in about 12 hrs, which thus represents a minimum time for bioavailable Fe to be utilised. Note that sediment grains (with attached nanoparticulates) will normally be removed during filtration and thus the measurement of 'dissolved' Fe (operationally defined by membrane filtration) may significantly under-estimate the amounts of bioavailable Fe.

Culture studies with marine diatoms [28,29] have shown that freshly-prepared, poorly-ordered Fe oxyhydroxides (primarily ferrihydrite with grain-sizes 2–16 nm) can supply Fe for cell growth but more crystalline phases (akaganeite, goethite and hematite) are not bioavailable. It is suggested [28] that bioavailability decreases with increasing thermodynamic stability. Thus the bioavailability of Fe decreases as poorly-ordered 2- and 6-line ferrihydrite lose water and age to their more crystalline and more stable counterparts (hematite and goethite). Aging and recrystallisation may occur in both oxic and anoxic environments but is slower at low temperatures [30,31]. The half-life for the conversion of 2-line ferrihydrite to goethite at 25°C decreases with increasing pH and is ~100 days at pH 7 [32]. Studies on the bioavailability of natural colloids collected from coastal and open ocean environments found intracellular uptake rates of 5–10% for colloidal Fe (1 nm-0.2 μm) by marine diatoms over a 12 hour period [33]. Bioavailability may also be enhanced by photochemical reactions [34-36] and protozoan grazing [37]. Most of these studies indicate that amorphous or poorly-crystalline ferrihydrite in the nanometer size range is at least partially bioavailable, and this is the phase most readily extracted by ascorbate (see above). Thus our measurements of FeA roughly correspond to the bioavailable Fe found in ferrihydrite and natural colloidal material. In the following estimates we make the conservative assumption that 5–10% of the ascorbate Fe is bioavailable, based on measurements of colloidal Fe uptake [33] and assuming a minimum time of 12 hrs for passage of attached nanoparticles through the surface water column.

### Bioavailable Fe Sources in the Southern Oceans

There are four main pathways by which icebergs may supply bioavailable Fe to the Southern Ocean (here defined as the area of 2.03 × 10^7 ^km^2 ^which lies south of 60°S). First, ice may contain dissolved Fe. Truly dissolved Fe is assumed to be bioavailable although the bioavailability of dissolved iron complexed with organic ligands varies between different phytoplankton [38]. Second, nanoparticulate Fe may be transferred to seawater when iceberg-hosted sediments are released by melting. Nanoparticulate Fe oxyhydroxides are ~2 orders of magnitude more soluble than their macroscopic counterparts [26] and solubility may be further enhanced by photoreduction and grazing (see above). The rates at which nanoparticulate Fe oxyhydroxides dissolve vary with mineralogy, crystallinity and surface area (in turn dependent on aging) but rapid dissolution of fresh nanoparticulates will supply bioavailable Fe to surface waters, whilst the slower dissolution of more aged and refractory oxides and oxyhydroxides (i.e., goethite, hematite) may supply Fe to deep waters. Third, nanoparticulate Fe may be dissolved by ice melt and added to seawater. Our ice melts have pH 6.0–7.5 and calculated concentrations of Fe^3+ ^species in equilibrium with Fe(OH)_3 _in this ice melt (1.2 to 17 nM) are similar to, or higher than (a) dissolved Fe concentrations in our icemelt (Table 1) and (b) dissolved iron concentrations [10] in the Pacific and Indian sectors of the Southern Oceans (~0.1 nM) and the Weddell Sea (~1 nM). Fourth and finally, atmospheric dust trapped in icebergs may have been subjected to cloud processing. Cloud droplets are acidic and successive cycles of dissolution and re-precipitation produce Fe phases that can be dissolved or remobilised following release by melting into seawater [4].

The data in Table 1 permit a comparison of the bioavailable iron fluxes derived from nanoparticulates in iceberg-hosted sediment, dissolved iron in melting icebergs and the dissolution of aeolian dust (Table 2). We will recalculate global data on Fe oxyhydroxide fluxes [9] to produce flux estimates for the Southern Ocean. Current estimates of iceberg calving from Antarctica are 2 Tm^3 ^yr^-1 ^of water equivalent [39] and small ice masses may contribute up to 0.5 Tm^3 ^yr^-1 ^[40]. Ice melting at this rate and containing 5–50 nM Fe (see above) supplies approximately 0.001–0.005 Tg yr^-1 ^of dissolved and bioavailable Fe (Table 2).

**Table 2:** Bioavailable Fe fluxes to the Southern Ocean

**Source**	**Mass flux**	**Bioavailable Fe concentrations**	**Fe flux Tg yr^-1^**
**Icemelt**	2.5 Tm^3 ^yr^-1^	5–50 nM	0.001–0.005
**Glaciers and Icebergs**	1250 Tg yr^-1^	0.009–0.012%	0.06–0.12
**Aeolian**	33 Tg yr^-1^	0.035–0.35%	0.01–0.13

The sediment content of icebergs is poorly known but a typical value is 0.5 kg m^-3 ^[9,41], a similar concentration to that in river water. Hence the flux of iceberg-hosted sediment to the Southern Ocean is 1250 Tg/yr. Iceberg sediment with a mean FeA content of 0.1% thus provides a flux of 1.2 Tg yr^-1 ^of FeA into the Southern Ocean. Assuming that 5–10% of FeA is biologically available to surface waters, we conservatively estimate a bioavailable, nanoparticulate Fe flux of 0.06–0.12 Tg yr^-1 ^(Table 2).

The debris content of non-basal ice in Antarctica is 0.2–2 g m^-3 ^[42,43], most of which was originally aeolian in origin. Aeolian dust collected by sea-ice may be responsible for the productivity increases associated with melting fronts [44-46]. However the direct supply of aeolian dust to the Southern Oceans is approximately 6% (27 Tg yr^-1^) of the global dust flux [3] which, together with the atmospheric dust (6 Tg yr^-1^) released by melting ice (see above) produces a combined dust flux of 33 Tg yr^-1 ^and a total Fe flux of 1.2 Tg yr^-1 ^(assuming a mean total Fe content of 3.5% [3]). Estimates of the proportion of Fe that can be solubilised from aeolian dust vary from 1–10% [3,47] but solubility increases with cloud processing, which in turn increases with transport distance (possibly solubilising as much as 40% of the total Fe in remote regions [48]). These data indicate a mean aeolian flux of 0.01 to 0.13 Tg yr^-1 ^of soluble, bioavailable Fe over the Southern Ocean as a whole, which is comparable to that conservatively estimated for icebergs and glaciers (Table 2).

The discussion has so far focused on the Southern Ocean as a whole but there are significant local enhancements of productivity in coastal regions [6,8] where iron limitation may be alleviated by supply from sea-ice melting and coastal sediments and, to a limited extent, by meltwater input. Aeolian dust collected by sea-ice may be responsible for the productivity increases associated with melting fronts [44-46] but dissolution of Fe from this source is unlikely to be any larger than that from aeolian dust supplied by icebergs (see above). The discharge of dissolved Fe by meltwaters from Antarctica has been estimated as 3 × 10^7 ^g yr^-1 ^[49], or approximately 1% of the dissolved Fe delivered by icemelt (Table 2). However coastal sediments may supply iron by several mechanisms, including diffusion from porewaters and sediment re-suspension [50] and are potentially a more prolific source. Measurements of the flux of dissolved Fe diffusing from porewaters in shelf sediments are generally low. Diffusive fluxes ranging up to 37 μg/cm^2^/yr have been observed [51-53] but higher values (100–400 μg/cm^2^/yr) have been estimated from modeling porewater profiles in Black Sea shelf sediments [54], North Sea sands and silts [55] and Arctic coastal sediments [56]. The magnitude of these diffusive fluxes is mainly controlled by the concentrations of dissolved Fe in porewaters and the thickness of the oxygenated surface layer of sediment, but is only weakly dependent on temperature [57]. These data indicate that diffusive recycling may be locally much more important than aeolian dust. The flux of total Fe supplied by dust to the Southern Ocean is <0.2 μg cm^-2 ^yr^-1 ^[45] so that the bioavailable Fe flux is < 0.02 μg cm^-2 ^yr^-1^, significantly less than measured or modelled values for diffusive fluxes from porewaters (but see [6]).

Furthermore diffusive fluxes may be greatly enhanced by bioirrigation and the physical reworking of shelf sediments, which promote mixing of iron-rich porewaters into overlying seawater [57]. The movement of partially grounded icebergs across the Antarctic shelf promotes significant, episodic physical re-working of shelf sediments that mixes dissolved, bioavailable Fe into shelf seawater. Diagenetic recycling (the sum of fluxes from diffusion, bioirrigation and physical reworking) may clearly supply more bioavailable Fe than dust in coastal regions. This bioavailable Fe is most likely to be used in near-shore areas rather than exported to the open ocean [50] and, consistent with this, enhanced productivity occurs in shelf and near-shore areas of the Southern Ocean [6]. However particulate hotspots derived from continental margin sediments have been found [58] more than 900 km from the coast. These particulates may represent coalesced nanoparticles (or nanoparticles attached to sediment grains) produced when Fe-rich sediment porewaters are mixed with seawater [9].

Atmospheric dust is clearly an important source of bioavailable Fe to the Southern Ocean but is spatially variable, increasing northwards and highest downwind of dry continental areas [57]. Considerable variability is also to be expected in the delivery of nanoparticulate Fe oxyhydroxides by the iceberg conveyor belt, but areas of high iceberg density and rapid iceberg melting, such as the Weddell Sea, are likely to be a prime focus. We believe that iceberg-hosted nanoparticulate Fe oxyhydroxides make a major contribution to the elevated Fe concentrations and increased productivity observed in the Weddell Sea and are also important in the Southern Ocean as a whole. It will be a challenging task to evaluate the impact of these temporally and spatially variable Fe fluxes in the Southern Ocean [12].

## Conclusion

Glacial and iceberg-hosted sediment contain Fe nanoparticles of ferrihydrite and goethite that are biogeochemically more reactive and more soluble than larger and more crystalline Fe oxyhydroxides. Nanoparticles enclosed in ice may be transported with minimal aging by icebergs away from coastal regions into the open ocean. A portion of this nanoparticulate Fe is likely to be bioavailable either directly or indirectly (following photochemical reactions or grazing by zooplankton). The most reactive portions of ferrihydrite and nanogoethite in glacial and iceberg-hosted sediments from Antarctica have been estimated by an ascorbate extraction which dissolves 0.10 ± 0.11% Fe from iceberg and glacial sediments. These are the first measurements of potentially bioavailable Fe on Antarctic ice-hosted sediments. These data can be combined with literature values for rates of iceberg calving from Antarctica and iceberg sediment contents to estimate the rates of nanoparticulate Fe delivery to the Southern Ocean as 0.06–0.12 Tg yr^-1^. This iceberg flux of bioavailable Fe to the Southern Ocean is comparable to that from aeolian dust (0.01–0.13 Tg yr^-1^), and considerably larger than the flux of dissolved Fe provided by icemelt (0.001–0.005 Tg yr^-1^).

We have shown that the present-day flux of glacial Fe oxyhydroxides to the Southern Ocean is sufficiently large that the dissolution of the tiny proportions of nanoparticulate Fe in this material may play a significant role in the delivery of bioavailable Fe; at least comparable to that from aeolian sources. A more comprehensive study of iceberg-hosted sediment is now required to ascertain the extent to which Fe oxyhydroxide nanoparticulates are present in icebergs and their geographical distribution, and examine their bioavailability experimentally. Identifying icebergs as a significant source of bioavailable Fe may shed new light on how the oceans respond to periods of atmospheric warming. The iceberg delivery of sediment containing nanoparticulate Fe during the Last Glacial Maximum (18000–21000 years ago) may have been sufficient to fertilize the increase in productivity required to drawdown CO_2 _to the levels observed in ice cores [9]. We speculate that, if icebergs mitigated against climate warming in the past, they may have the capacity to do so in the near-future.

## Competing interests

The authors declare that they have no competing interests.

## Authors' contributions

RR carried out the fieldwork in Antarctica, sampling and wet chemical analyses, and drafted the manuscript. LGB carried out the high resolution microscopy and provided nanoparticulate expertise. MT and ST supplied the glacial samples and provided the glaciological input to the manuscript. All the authors assisted in preparing the manuscript, and read and approved the final manuscript.
